# Supplementary material for: Identification of a modulator of the actin cytoskeleton, mitochondria, nutrient metabolism and lifespan in yeast
Source: Nat Commun. 2022 May 16;13:2706. doi: 10.1038/s41467-022-30045-9 (PMC9110415; doi:10.1038/s41467-022-30045-9)
Supplement: Supplementary file 1 — Supplementary Information [file 41467_2022_30045_MOESM1_ESM.pdf]

Supplementary information for

Identification of a modulator of the actin cytoskeleton, mitochondria, nutrient metabolism and lifespan in yeast

Cierra N. Sing[1,2], Enrique J. Garcia[1], Thomas G. Lipkin[1,3], Thomas M. Huckaba[1,4], Catherine A. Tsang[1], Arielle C. Coughlin[1], Emily J. Yang[1], Istvan R. Boldogh[1], Ryo Higuchi-Sanabria[1,2,5] and Liza A. Pon[1,2*]

*Corresponding Author: Liza A. Pon, Ph.D.

e-mail: lap5@cumc.columbia.edu

The following .pdf file includes Supplementary Figures 1 – 4.

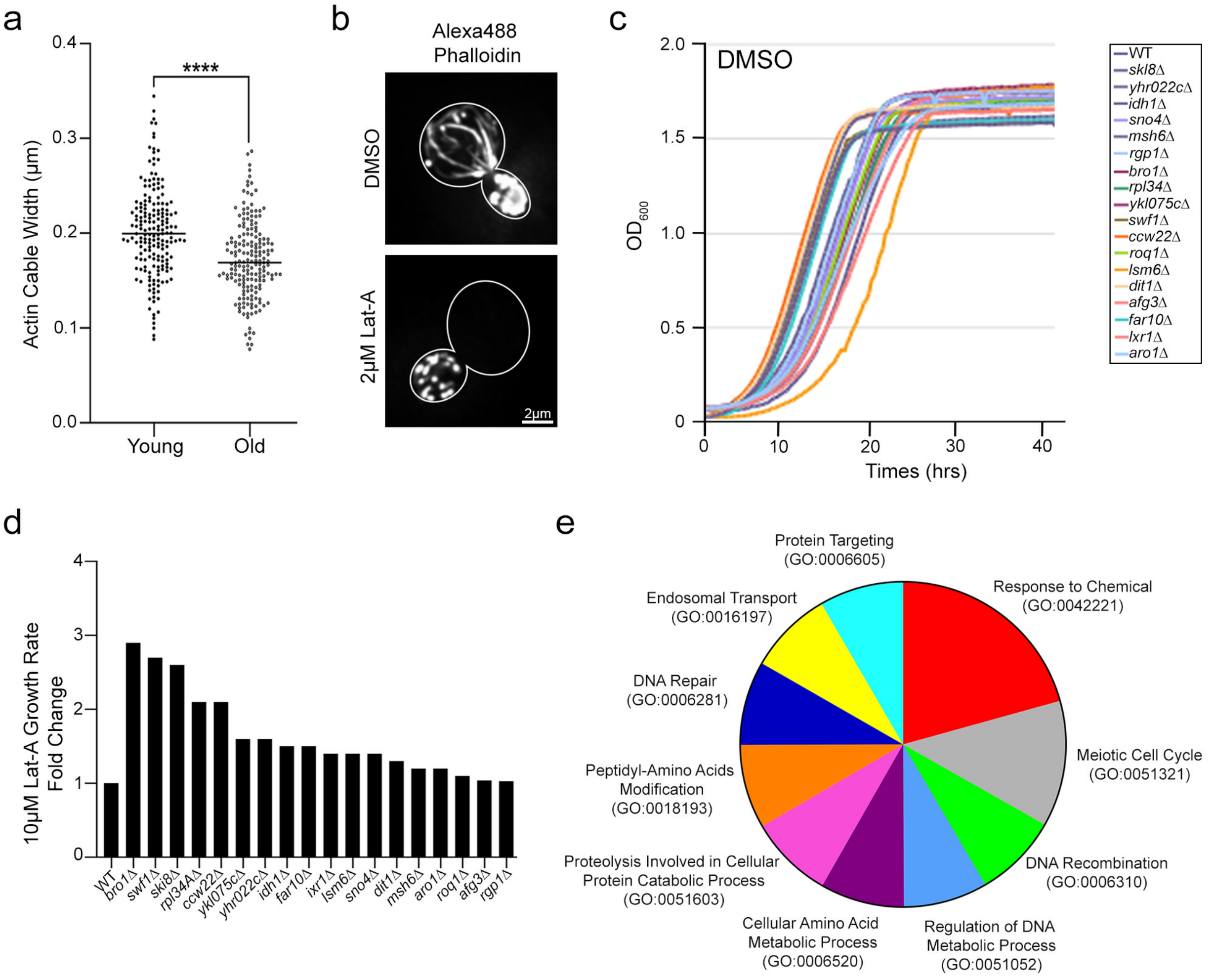

Supplementary Fig. 1: Phenotypic analysis of aging effects on actin cables and of the hits identified in the screen for actin cable modulators.

a, Quantification of apparent actin cable width in young and old WT cells. Data from representative trial. n of cells/strain: 183 (young) and 184 (old) cells. *p* value: $1.75e^{-10}$ (two-tailed non-parametric Mann-Whitney test). **b**, Representative images of Alexa488-phalloidin stained actin structures in mid-log phase WT cells treated with vehicle (DMSO) or 2 µM Lat-A for 20 mins. **c**, Growth curves of hits from the screen in the presence of vehicle (DMSO). **d,** Growth rate of deletion strains relative to WT cells upon treatment with 10 µM Lat-A. **e**, Gene ontology (GO) terms from the biological process Ontology of current annotated genes identified as hits in

the Lat-A screen determined using SGD GO SLIM. GO terms are shown as percent to the total ORFs for GO terms associated with 2 or more hits.

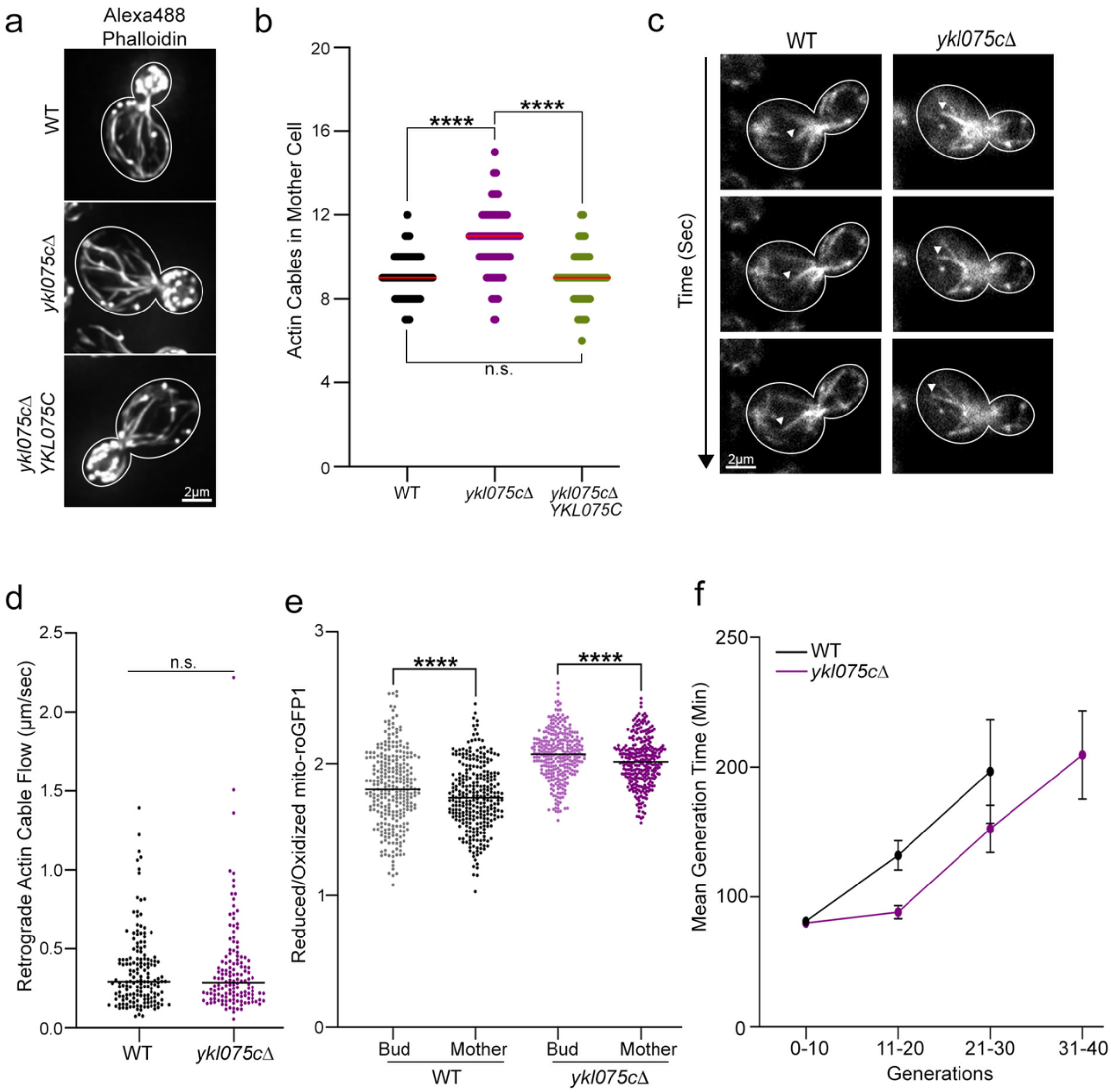

Supplementary Fig. 2: Phenotypic analysis of the actin cytoskeleton and mitochondrial quality in *ykl075c*Δ cells.

a, Representative images of Alexa488 phalloidin-stained F-actin of mid-log phase WT and *ykl075c*Δ cells and *ykl075c*Δ cells that express *YKL075C*. **b,** Quantification of actin cable abundance in WT, *ykl075c*Δ, and *ykl075c*Δ*YKL075C* cells. Combined *n* of cells/strain of 3 trials: 170 (WT), 187 (*ykl075c*Δ), 169 (*ykl075c*Δ*YKL075C*). *p* values determined by two-tailed non-parametric Kruskal-Wallis test: $<1.00e^{-15}$ (WT vs *ykl075c*Δ), >0.999 (WT vs. *ykl075c*Δ*YKL075C)*, and $<1.00e^{-15}$ (*ykl075c*Δ vs. *ykl075c*Δ*YKL075C*). **c**, Time-lapse frames

showing retrograde actin cable flow (RACF) visualized using Abp140p-GFP in mid-log phase WT and *ykl075c*Δ cells. Arrowheads point to a motile actin cable. **d**, Quantification of RACF rates in WT and *ykl075c*Δ cells. Combined *n* of cells/strain of 3 trials: 160 (WT) and 140 (*ykl075c*Δ). n.s. – not significant (two-tailed non-parametric Mann-Whitney test). **e**, Reduced:oxidized mito-roGFP1 ratios of mitochondria in mother cells and buds of mid-log phase WT and *ykl075c*Δ cells. Combined *n* of cells/strain of 3 trials: 284 (WT bud and mother), 300 (*ykl075c*Δ bud and mother). *p* values $6.05e^{-12}$ (Bud_{WT} vs. $Mother_{WT}$) and $1.94e^{-09}$ ($Bud_{ykl075c\Delta}$ vs. $Mother_{ykl075c\Delta}$). *p* values were determined by two-tailed Wilcoxon matched-pair test. **f**, Quantification of mean generation time (time between emerging consecutive buds from the same mother) of WT and *ykl075c*Δ cells during RLS analysis. Greater than 35 cells/strain/trial were analyzed for *n* of 3 trials. Error bars: SEM.

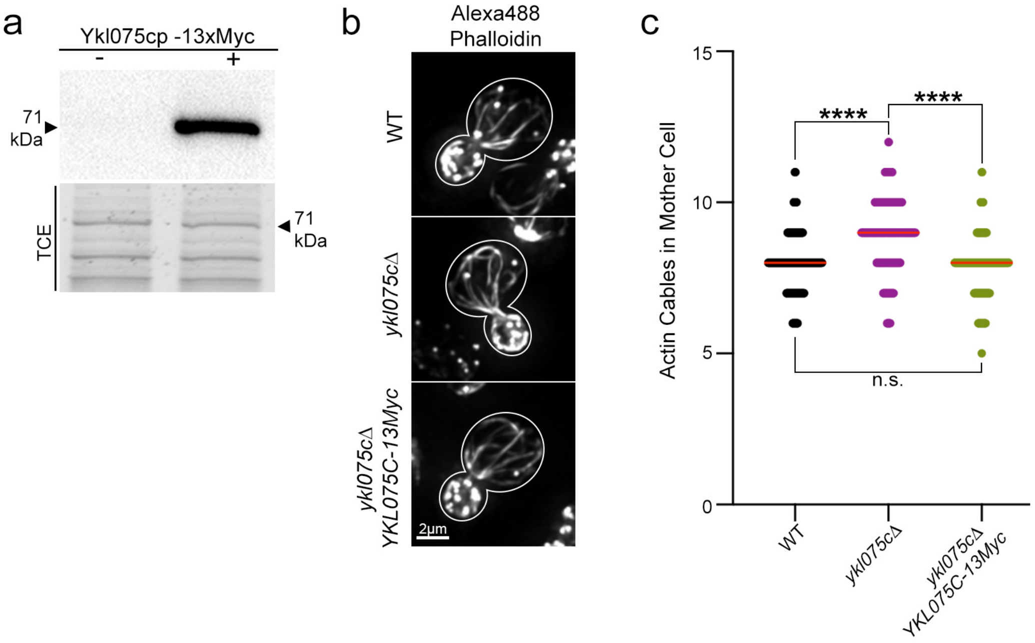

Supplementary Fig. 3: Analysis of Ykl075cp-13Myc for cellular localization.

a, Western blot of Ykl075cp (Aan1p) tagged at its C-terminus with 13 copies of the Myc epitope. TCE, total protein loading control. Data is representative of *n* of 3 trials. **b,** Representative images of Alexa488 phalloidin-stained F-actin of mid-log phase WT and *ykl075c*Δ cells, and *ykl075c*Δ that express *YKL075C-13Myc*. **c**, Quantification of actin cable abundance in WT, *ykl075c*Δ, and *ykl075c*Δ*YKL075C-13Myc* cells. Combined *n* of cells/strain of 3 trials: 211 (WT), 209 (*ykl075c*Δ), 192 (*ykl075c*Δ*YKL075C-13Myc*). *p* values <1.00e^{-15} (WT vs. *ykl075c*Δ), 8.45e^{-01} (WT vs. *ykl075c*Δ*YKL075C-13Myc*) <1.00e^{-15} (*ykl075c*Δ vs. *ykl075c*Δ*YKL075C-13Myc*). *p* values determined by two-tailed non-parametric Kruskal-Wallis test**.**

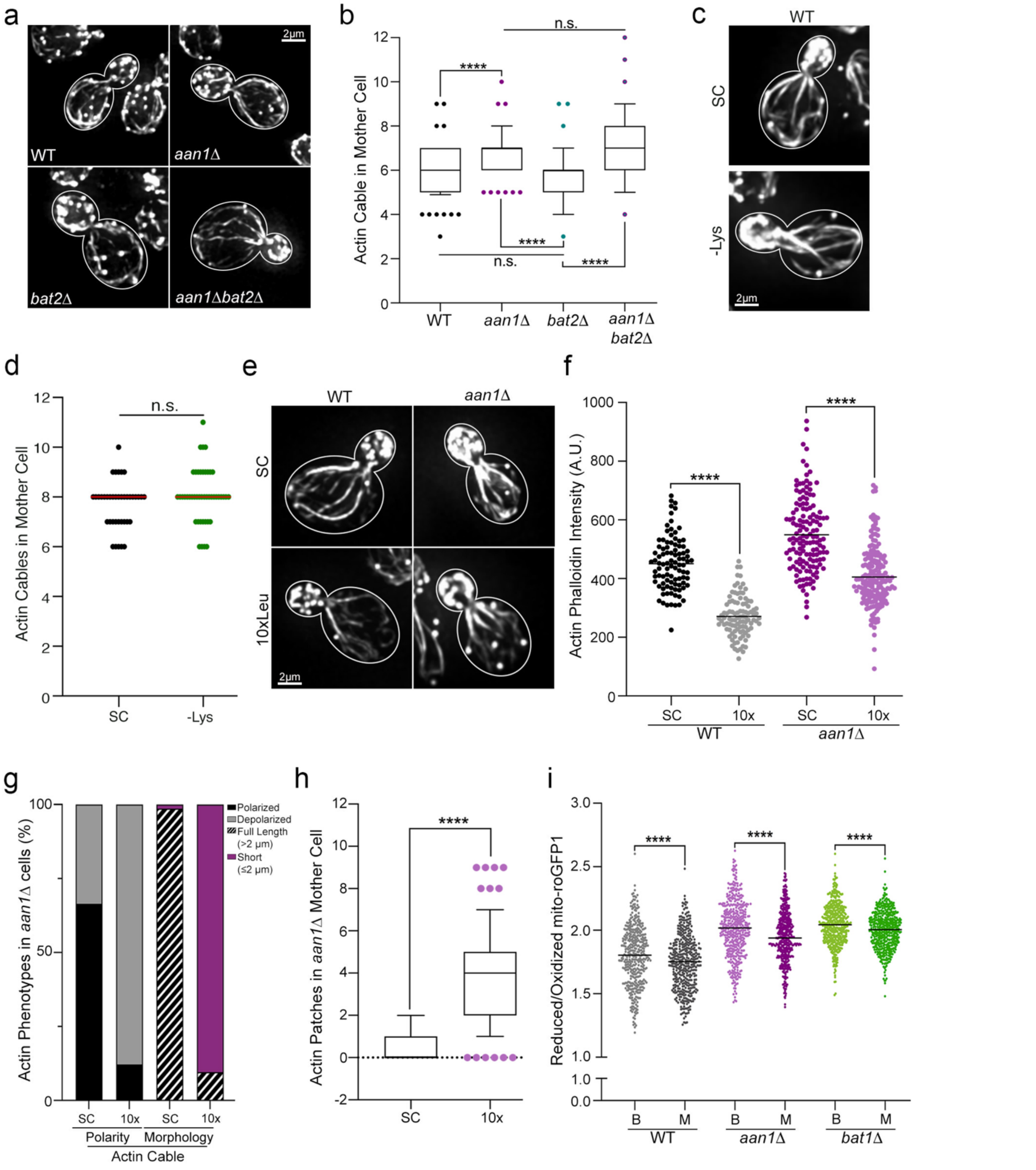

Supplementary Fig. 4: *AAN1* function in modulating actin cables through effects on BCAA homeostasis.

a, Representative images of Alexa488 phalloidin-stained F-actin of mid-log phase WT, *aan1(ykl075c)*Δ, *bat2*Δ, or *aan1*Δ *bat2*Δ cells. **b**, Quantification of actin cable abundance in WT,

*aan1*Δ, *bat2*Δ, or *aan1*Δ *bat2*Δ cells are plotted in a box and whisker plot. The center band represents the median; the box indicates middle quartiles; whiskers extend to the 10th and 90th percentiles. Data from representative trial. n of cells/strain: 78 (WT), 70 (*aan1*Δ), 88 (*bat2*Δ), 68 (*aan1*Δ*bat2*Δ). *p* value: $6.31e^{-06}$ (WT vs. *aan1*Δ), >0.999 (WT vs *bat2*Δ), $1.71e^{-07}$ (*aan1*Δ vs *bat2*Δ), >0.999 (*aan1*Δ vs. *aan1*Δ*bat2*Δ), and $3.58e^{-08}$ (*bat2*Δ vs. *aan1*Δ*bat2*Δ). *p* value determined by two-tailed non-parametric Kruskal-Wallis test. **c**, Representative images of Alexa488 phalloidin-stained F-actin of mid-log phase WT cells grown in synthetic complete (SC) medium or SC without lysine (-Lys) for 30 min. **d**, Quantification of actin cable abundance in the presence or absence of lysine. Combined n of cells/strain/group: 129 (WT_{SC}), and 135 (WT_{-Lys}). *p* values: $3.65e^{-01}$ (WT SC vs. -Lys). Data values were generated from 3 trials and analyzed using a two-tailed non-parametric Mann-Whitney test (n.s., not significant). **e**, Representative images of AlexaFluor488-phalloidin stained actin cytoskeleton of mid-log phase WT and *aan1*Δ cells grown in synthetic complete (SC) medium or SC with leucine supplementation. Scale bar, 2 µm. **f**, Quantification of F-actin phalloidin mean signal intensity in mother cells of WT and *aan1*Δ strains. Data from representative trial. n of cells/strain: 95 (WT in SC), 100 (WT in 10xLeu), 143 (*aan1*Δ in SC), and 183 (*aan1*Δ in 10xLeu). *p* value were determined by two-taild non-parametric Kruskal-Wallis test: <$1.00e^{-15}$ (WT_{SC} vs. WT_{10xLeu}) and <$1.00e^{-15}$ *(aan1*$Δ_{SC}$ *vs aan1*$Δ_{10xLeu}$ (non-parametric Kruskal-Wallis test). **g**, The actin cytoskeleton phenotypes in mid-log phase *aan1*Δ cells grown in SC (n = 146 cells) and SC with 10x leucine (n = 114 cells) stained with AlexaFluor488-phalloidin. Full length actin cables are ≥ 2 µm and typically extend from the bud to the tip of the mother cell distal to the bud. **h**, Quantification of actin patches within mother cells of *aan1*Δ cells grown in SC (n = 143 cells) and SC with 10x leucine (n = 117 cells). Data is represented in a box and whisker plot. The center band represents the median; the box indicates middle quartiles; whiskers extend to the 10th and 90th percentiles. *p* value: <$1.00e^{-15}$ (two-tailed Mann-Whitney test). **i**, Reduced:oxidized mito-roGFP1 ratios of mitochondria in mother and buds of mid-log phase WT, *aan1*Δ, and *bat1*Δ cells. Combined n of

3 trials: 419 (WT bud and mother), 461 (*aan1*Δ bud and mother), and 486 (*bat1*Δ bud and mother). *p* value was determined by two-tailed Wilcoxon matched-pair test: $4.08e^{-09}$ ($Mother_{WT}$ vs Bud_{WT}), $<1.00e^{-15}$ ($Mother_{aan1\Delta}$ vs. $Bud_{aan1\Delta}$), and $2.72e^{-13}$ ($Mother_{bat1\Delta}$ vs. $Bud_{bat1\Delta}$).
