# Supplementary material for: Identification of a modulator of the actin cytoskeleton, mitochondria, nutrient metabolism and lifespan in yeast
Source: Nat Commun. 2022 May 16;13:2706. doi: 10.1038/s41467-022-30045-9 (PMC9110415; doi:10.1038/s41467-022-30045-9)
Supplement: Supplementary file 6 — Reporting Summary [file 41467_2022_30045_MOESM6_ESM.pdf]

nature research

Corresponding author(s): Liza Pon

Last updated by author(s): 02-07-2022

Reporting Summary

Nature Research wishes to improve the reproducibility of the work that we publish. This form provides structure for consistency and transparency in reporting. For further information on Nature Research policies, see our Editorial Policies and the Editorial Policy Checklist.

Statistics

For all statistical analyses, confirm that the following items are present in the figure legend, table legend, main text, or Methods section.

n/a	Confirmed	
☐	☒	The exact sample size (*n*) for each experimental group/condition, given as a discrete number and unit of measurement
☐	☒	A statement on whether measurements were taken from distinct samples or whether the same sample was measured repeatedly
☐	☒	The statistical test(s) used AND whether they are one- or two-sided<br>*Only common tests should be described solely by name; describe more complex techniques in the Methods section.*
☐	☒	A description of all covariates tested
☐	☒	A description of any assumptions or corrections, such as tests of normality and adjustment for multiple comparisons
☐	☒	A full description of the statistical parameters including central tendency (e.g. means) or other basic estimates (e.g. regression coefficient) AND variation (e.g. standard deviation) or associated estimates of uncertainty (e.g. confidence intervals)
☐	☒	For null hypothesis testing, the test statistic (e.g. *F*, *t*, *r*) with confidence intervals, effect sizes, degrees of freedom and *P* value noted<br>*Give P values as exact values whenever suitable.*
☒	☐	For Bayesian analysis, information on the choice of priors and Markov chain Monte Carlo settings
☐	☒	For hierarchical and complex designs, identification of the appropriate level for tests and full reporting of outcomes
☒	☐	Estimates of effect sizes (e.g. Cohen's *d*, Pearson's *r*), indicating how they were calculated

Our web collection on statistics for biologists contains articles on many of the points above.

Software and code

Policy information about availability of computer code

Data collection	Images data collected on:<br>1. Axioskop 2 microscope equipped with a 100x/1.4 Plan-Apochromat objective (Zeiss, Thornwood NY), an Orca-ER CCD camera (Hamamatsu Corporation, Bridgewater NJ), a pE-4000 LED illumination system (coolLED, Andover UK,), controlled by Nikon NIS Elements 4.60 Lambda software<br>2. Structured illumination microscope (N-SIM S, Nikon) equipped with a 100x/1.49 oil-immersion objective lens (Nikon Instruments, Melville NY), an EMCCD Camera (iXon, Andor Technology Ltd, Belfast Ireland) and NIS Elements software (Nikon Instruments, Melville NY).
Data analysis	Imaging analysis data was processed using Volocity Software 5.5 (Quorum Technologies). Statistical analysis was performed with Prism 9 (Graphpad). No additional software or script packages were used to analyze and/or processing images aside from the software that was listed.

For manuscripts utilizing custom algorithms or software that are central to the research but not yet described in published literature, software must be made available to editors and reviewers. We strongly encourage code deposition in a community repository (e.g. GitHub). See the Nature Research guidelines for submitting code & software for further information.

Data

Policy information about availability of data

All manuscripts must include a data availability statement. This statement should provide the following information, where applicable:

- Accession codes, unique identifiers, or web links for publicly available datasets
- A list of figures that have associated raw data
- A description of any restrictions on data availability

Data generated from RNAseq dataset reported in this study is available to the public under the accession code (GSE174157) and in this study source data file. Gene ontology analysis was gathered from Yeastract+ database (http://yeastract.com/). All primer designs were based on reference genome from the S. cerevisiae

genome database (https://www.yeastgenome.org/) and are available in the Supplementary Information. All analyzed data generated from this study are included in this published article.

Field-specific reporting

Please select the one below that is the best fit for your research. If you are not sure, read the appropriate sections before making your selection.

☒ Life sciences ☐ Behavioural & social sciences ☐ Ecological, evolutionary & environmental sciences

For a reference copy of the document with all sections, see nature.com/documents/nr-reporting-summary-flat.pdf

Life sciences study design

All studies must disclose on these points even when the disclosure is negative.

Sample size	Replicative lifespan analysis involved > 35 cells for statistical significance.<br>Imaging analysis cell sample size: >45 cells per condition per trial.
Data exclusions	No data exclusions were made aside from identifying the outliers.
Replication	All experimental findings were reliably reproduced with biological replicates.
Randomization	All strains from this study are isogenic, thus any genetic manipulation or chemical treatment is intrinsically randomized.
Blinding	All experiments were not blinded during data collection. Blinding is not applicable to these studies given the nature of the experimental design.

Reporting for specific materials, systems and methods

We require information from authors about some types of materials, experimental systems and methods used in many studies. Here, indicate whether each material, system or method listed is relevant to your study. If you are not sure if a list item applies to your research, read the appropriate section before selecting a response.

Materials & experimental systems

n/a	Involved in the study	
☐	☒	Antibodies
☒	☐	Eukaryotic cell lines
☒	☐	Palaeontology and archaeology
☒	☐	Animals and other organisms
☒	☐	Human research participants
☒	☐	Clinical data
☒	☐	Dual use research of concern

Methods

n/a	Involved in the study	
☒	☐	ChIP-seq
☒	☐	Flow cytometry
☒	☐	MRI-based neuroimaging

Antibodies

Antibodies used	1. Mouse monoclonal clone 9E10 anti-Myc primary antibody, used in wb, produced by Developmental Studies Hybridoma Bank, University of Iowa<br>2. Goat anti-Mouse IgG Alexa Fluor Plus 488 Secondary Antibody, used in immunofluorescence, Themofisher, Ref A32723<br>3. Polyclonal anti-rabbit Phospho-S6 Ribosomal Protein (Ser235/236) Antibody, used in wb, Cell Signaling, Ref 2211,
Validation	1. Mouse monoclonal clone 9E10 anti-Myc primary antibody, Developmental Studies Hybridoma Bank, University of Iowa manual can be found at 'https://dshb.biology.uiowa.edu/9E-10'.<br>2. Goat anti-Mouse IgG Alexa Fluor Plus 488 Secondary Antibody, Themofisher, Ref A32723, manual can be found at 'https://www.thermofisher.com/antibody/product/Goat-anti-Mouse-IgG-H-L-Highly-Cross-Adsorbed-Secondary-Antibody-Polyclonal/A32723'.<br>3. Polyclonal anti-rabbit Phospho-S6 Ribosomal Protein (Ser235/236) Antibody, Cell Signaling, Ref 2211, manual can be found at 'https://www.cellsignal.com/products/primary-antibodies/phospho-s6-ribosomal-protein-ser235-236-antibody/2211'.
